# Metabolism in Human Mesenchymal Stromal Cells: A Missing Link Between hMSC Biomanufacturing and Therapy?

**DOI:** 10.3389/fimmu.2019.00977

**Published:** 2019-05-08

**Authors:** Xuegang Yuan, Timothy M. Logan, Teng Ma

**Affiliations:** ^1^Department of Chemical and Biomedical Engineering, Florida State University, Tallahassee, FL, United States; ^2^Institute of Molecular Biophysics, Florida State University, Tallahassee, FL, United States; ^3^Department of Chemistry and Biochemistry, Florida State University, Tallahassee, FL, United States

**Keywords:** MSCs (mesenchymal stromal cells), immunomodulation, metabolic plasticity, biomanufacturing, therapeutic potentials

## Abstract

Human mesenchymal stem cells (hMSCs) are the most commonly-tested adult stem cells in cell therapy. While the initial focus for hMSC clinical applications was to exploit their multi-potentiality for cell replacement therapies, it is now apparent that hMSCs empower tissue repair primarily by secretion of immuno-regulatory and pro-regenerative factors. A growing trend in hMSC clinical trials is the use of allogenic and culture-expanded cells because they are well-characterized and can be produced in large scale from specific donors to compensate for the donor pathological condition(s). However, donor morbidity and large-scale expansion are known to alter hMSC secretory profile and reduce therapeutic potency, which are significant barriers in hMSC clinical translation. Therefore, understanding the regulatory mechanisms underpinning hMSC phenotypic and functional property is crucial for developing novel engineering protocols that maximize yield while preserving therapeutic potency. hMSC are heterogenous at the level of primary metabolism and that energy metabolism plays important roles in regulating hMSC functional properties. This review focuses on energy metabolism in regulating hMSC immunomodulatory properties and its implication in hMSC sourcing and biomanufacturing. The specific characteristics of hMSC metabolism will be discussed with a focus on hMSC metabolic plasticity and donor- and culture-induced changes in immunomodulatory properties. Potential strategies of modulating hMSC metabolism to enhance their immunomodulation and therapeutic efficacy in preclinical models will be reviewed.

## Background

Human mesenchymal stem or stromal cells (hMSCs) are the most commonly-tested adult stem cells in experimental cell therapy and have been used in more than 50% of clinical trials using stem cells since 2000. Clinically, the most beneficial aspects of hMSCs are their multilineage differentiation for damage tissue replacement and their ability to empower tissue repair by secretion of immuno-regulatory and pro-regenerative factors. Clinical applications of hMSC-based therapy initially exploited their multi-potentiality but increasingly focused on their secretion of immunomodulatory and trophic factors. In this immunoregulatory scenario, hMSCs promote tissue regeneration by coordinating an anti-inflammatory response through communication with the host's inflammatory microenvironment, making hMSC logical candidates for the treatment of immune disorders and inflammatory diseases. In contrast to the promising results from preclinical studies and small-scale clinical trials, the clinical outcomes using manufactured hMSC have been inconsistent and suboptimal (1–3). The close scrutiny of the discrepant outcome from these studies suggests that culture expansion, cryopreservation, and inappropriate delivery routes and dosage, are major factors that adversely influence hMSC's therapeutic efficacy (1, 4). For example, in graft-vs.-host disease, 1-year survival for patients receiving hMSC at passages 1–2 was 75% in contrast to 21% using hMSC at passages 3–4 (5). In clinical application, hMSC therapeutics are often cryobanked as “off-the-shelf” products prior to transfusion. However, cryopreservation is known to reduce hMSC immunomodulatory properties as a result of cellular stress such as cellular acidosis and metabolic uncoupling induced during freezing and thaw cycles (6, 7). It is worth noting that many of these functional changes are not readily reflected in the assessment of the minimal criterial and potency assays, suggesting the need for the identification of additional surrogate markers and regulatory pathways that can be readily assessed, and modulated to restore hMSC therapeutic potency.

This immunoregulatory function is achieved through rapid hMSC phenotype polarization and sustained production of immunoregulatory factors in response to inflammatory stimuli, which requires cellular plasticity and metabolic fitness to enable this response (8). The metabolic fitness of hMSCs is dependent on donor age and morbidity, and can be significantly altered by culture conditions imposed on the cells during *in vitro* expansion. Each of these factors, and probably other, currently unknown factors, can reduce hMSC immunomodulatory capacity and, therefore, reduce their therapeutic potency. A typical clinical dose of hMSCs is on the order of tens- to hundreds- of millions of cells per patient and, with hMSCs being approved for use in an ever-expanding number of clinical indications, it is estimated 300 trillion (300 × 10^12^) hMSCs will be needed annually by 2030 (9, 10). Current engineering protocols isolate hMSCs from adult donors and expand them under nutrient-rich conditions that significantly promote proliferation but ultimately and inadvertently reduce stemness and therapeutic potency. To compensate for the culture-induced decline of hMSC therapeutics potency, non-genetic preconditioning such as hypoxia pretreatment or 3D culture has demonstrated significant potential in restoring hMSC properties (1). To better preserve hMSC property during cryopreservation, hydrogen peroxide preconditioning has also been shown to enhance adipose derived stem cells (ASCs) resistance and survival under oxidative stress (7). While many of these preconditioning strategies have demonstrated effectiveness in restoring hMSC functional properties, the regulatory mechanism remains to be fully understood for widespread implementation in hMSC manufacturing and translation (11).

Among the core pathways to improving hMSC function, metabolism has emerged as an important hub. In their native environment, hMSCs are present in a quiescent state characterized by low proliferation and high multi-potentiality, which is maintained throughout adult life. In this state, stem cells appear to be primarily glycolytic, with “young” mitochondria maintained by active autophagy and mitophagy (12, 13). However, numerous studies show that transferring hMSC into the nutrient-rich artificial culture environment that promotes rapid proliferation reconfigures their central energy metabolism to become significantly more dependent on oxidative phosphorylation (OXPHOS) to support the rapid proliferation (14, 15). The high OXPHOS-fueled metabolic profile also results in accumulation of cytotoxic metabolic byproducts, including reactive oxygen species (ROS) (16), that reduce the basal autophagy and mitophagy rate while simultaneously increasing the fraction of senescent cells with reduced clinical potency (17). Similar metabolic alterations have been reported for hMSC undergoing large-scale expansion in bioreactor systems (18). The influence of these metabolic changes on hMSC functional properties has just begun to be revealed. Beyond providing cells with building blocks and energy source to power cellular processes, the energy metabolism and intermediate metabolites play important roles in shaping cellular functional properties (19). Therefore, a clearer understanding of how hMSC metabolism is affected by long-term culture and how specific metabolic states impact immunomodulatory function may be the missing link between engineering practices for expansion and consistent, predictable outcomes in hMSC-based therapies. This review focuses on the role of energy metabolism in regulating hMSC immunomodulatory properties and discusses its implication for hMSC large-scale manufacturing and therapy. The specific characteristics of hMSC metabolism, culture-induced metabolic changes, and the metabolism underpinning hMSC's immunomodulatory properties will be discussed. We will also review and discuss recent studies on hMSC metabolic modulation of their immunomodulatory properties and therapeutic efficacy.

## hMSCs Metabolic Plasticity and Culture-Induced Metabolic Changes

**Figure 1:**
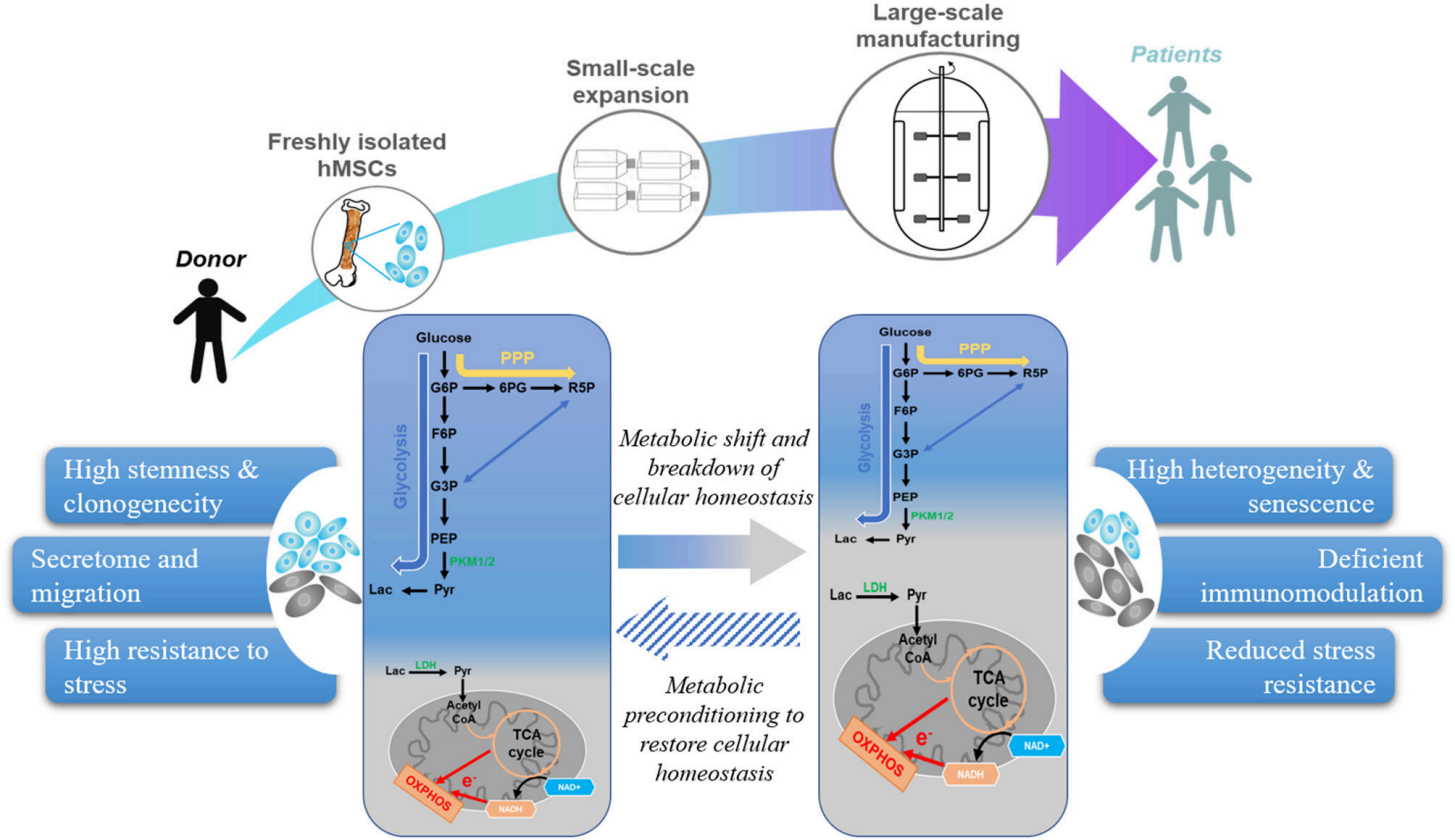
Current hMSC manufacturing practices lead to metabolic shift that reduces therapeutic properties. hMSC manufacturing utilizes freshly isolated hMSCs and expand under artificial environment to obtain sufficient cell number for clinical application. However, external stresses during replicative expansion, and cryopreservation shift hMSC metabolism from glycolysis toward OXPHOS, which increases senescent subset and contributes to a breakdown of cellular homeostasis. Metabolic preconditioning targeting specific pathways can restore hMSC cellular homeostasis and enhance their therapeutic potency.

Once viewed as a mere consequence of the cellular state, energy metabolism not only provides energy and substrates for cell growth but is also intimately linked to cell signaling and control of cell fate (16, 20, 21). As depicted in Figure 1, freshly isolated hMSCs mainly compressed a clonogenic subset with high glycolytic activity. Expansion under currently adopted engineering protocols reduces the fraction of clonogenic/glycolytic cells and increases the fraction of mature/OXPHOS-based cells. In fact, this phenotypic and metabolic heterogeneity exists even at the clonal level of hMSCs (22). It has been suggested that changes in the microenvironment and accumulated replicative stress experienced during extended *in vitro* expansion accelerate hMSC cellular and metabolic heterogeneity by reconfiguring energy metabolism (21, 22). As mentioned above, hMSCs exhibit a quiescent, glycolytic phenotype in a hypoxic *in vivo* niche such as bone marrow (16, 21). This particular metabolic state may serve to preserve hMSC cellular homeostasis by minimizing ROS production (16, 21, 23), because the high glycolytic flux is also cytoprotective due to increased generation of antioxidant precursors from the pentose phosphate pathway (PPP) (24). Upon removal from this hypoxic, *in vivo* niche and transfer to the oxygenated, nutrient-rich environment, this quiescent, glycolytic phenotype is no longer of benefit to hMSC (11, 22). During early passages, hMSCs still maintain to an aerobic glycolysis profile, despite its low efficiency for ATP production (25). However, protracted expansion of hMSCs under this nutrient-rich environment induces a metabolic shift from glycolysis toward OXPHOS (22, 26). This metabolic shift is associated with increased coupling between glycolysis and TCA cycle and significantly increased production of ROS and dysfunctional mitochondria (21). A consequence of this culture-induced metabolic shift is a breakdown of cellular homeostasis, characterized by reduced autophagy/mitophagy activity and increased senescence (22, 27–29). A recent proteomic analysis identified several proteins involved in energy metabolism, mitochondrial dysfunction, OXPHOS, and nuclear factor erythroid 2–related factor 2 -mediated oxidative stress response as being among the top canonical pathways that are altered in large-scale production of hMSC in bioreactor system (18). In addition, intrinsic biological factors such as donor disease or chronological age also alter hMSC metabolic profile. For example, hMSCs from obese donors have a higher number of defective mitochondria with reduced dependence on glycolysis and an altered metabolic profile compared to cells obtained from healthy controls (30, 31). hMSCs from aged donors also exhibit greater population heterogeneity with lower mitochondrial-to-cytoplasm area ratio, reduced level of manganese superoxide dismutase (MnSOD) expression, and accumulated ROS compared with cells obtained from young donors (32). Furthermore, hMSCs from donors with age-related atherosclerosis exhibited impaired mitochondrial function that also contributed to metabolic alterations compared to cells obtained from healthy, young donors (33). Culture-based interventions aimed at restoring the mitochondrial function of atherosclerotic-hMSCs by treatment with ROS scavengers effectively restored their immunosuppressive ability to that of healthy donors (33). This study directly linked metabolic alteration due to donor morbidity to reduced hMSC therapeutic potency and demonstrated that metabolic treatment can restore hMSCs to a state capable of delivering the desired therapeutic outcomes. In contrast, the extent of influence of culture-induced metabolic changes on hMSC therapeutic potency is largely unknown because few studies have characterized the metabolic profiles of hMSC used in therapeutic applications. Beyond supplying energy and anabolic production of macromolecules, metabolic circuits engage genetic programs to regulate cellular events and phenotypic and functional properties, reflecting the metabolic substrate, specific pathways, and environmental conditions (23). Although large scale hMSC biomanufacturing often entails significant changes of culture conditions such as media composition and substrates (e.g., sugar *vs*. fatty acids), supplements (e.g., fetal bovine serum *vs*. platelet lysate), expansion protocols (e.g., multi-well plates vs. bioreactor), and cryopreservation (e.g., cryogen and freeze-thaw cycles), few studies have elucidated the influence of these changes on hMSC functional properties and clinical outcome (1, 6, 34–38).

Preclinical studies on the mechanistic connections between metabolism and hMSC phenotype provide specific molecular targets and pathways that can be modulated to maintain, or remodel metabolic profiles of culture expanded hMSCs. For example, hypoxia culture has been widely used in hMSC expansion as it better preserves the clonogenic subset during hMSC expansion by maintaining glycolysis and suppressing TCA cycle and OXPHOS activity (15, 26, 39, 40), most likely through activation of hypoxia-inducible factor (HIF) related genes (41). As a consequence of the glycolytic metabolic profile, hypoxia culture also activates hMSC autophagy while reducing senescence and preserving hMSC functional properties by maintaining cellular homeostasis *in vitro* (40, 42). However, hMSCs are not only sensitive to the absolute oxygen level but also to the fluctuation in oxygen tension, which significantly complicates implementing hypoxia as an engineering protocol for maintaining stem cell properties in large scale manufacturing (43, 44). More recently, the benefits of hMSC hypoxia culture have been recapitulated at ambient oxygen tensions by treatment of small molecule modulators that target specific metabolic regulatory pathways identified in hypoxia studies. Since HIF is a downstream effector of mTOR, inhibition of Akt/mTOR signaling pathway with rapamycin and LY294002 reduced mitochondrial activity, and glycolysis–TCA coupling, prevented culture-induced senescence (20, 45). These studies highlight the importance for a balance between AKT/mTOR activity and intracellular signaling for maintaining glycolytic metabolism to preserve stem cell functions. Non-hypoxic stabilization of HIF-1α using hypoxia mimetics such as desferoxamine (DFO), ciclopirox olamine, the HIF-prolyl hydroxylase (PHD) inhibitor FG-4497, or cobalt chloride (CoCl2) have also been shown to overcome the challenge of controlling ambient oxygen tension to effectively maintain MSC properties (45–47).

hMSCs have intriguing properties of self-assembly into three-dimensional (3D) aggregates mediated by cell-cell and cell-ECM interaction, which better preserve hMSC phenotypic properties compared to their 2D counterparts (48). The benefits of 3D aggregation culture in preserving hMSC stemness and enhancing secretion of immunomodulatory cytokines can also be attributed to aggregation-induced metabolic reconfiguration, which inhibits mitochondrial activity and increases glycolysis with increased anaplerotic flux (28, 48–50). While the metabolic reprograming in 3D aggregates has been commonly attributed to oxygen diffusion limitation, our recent studies reveal that actin-mediated cellular compaction activates PI3K/Akt pathway and induces metabolic shift toward glycolysis (48, 51), highlighting energy metabolism as a signaling hub in regulating hMSC functions during *in vitro* culture.

## The Role of Metabolism in the hMSC Immune Response and Immunomodulation

**Figure 2:**
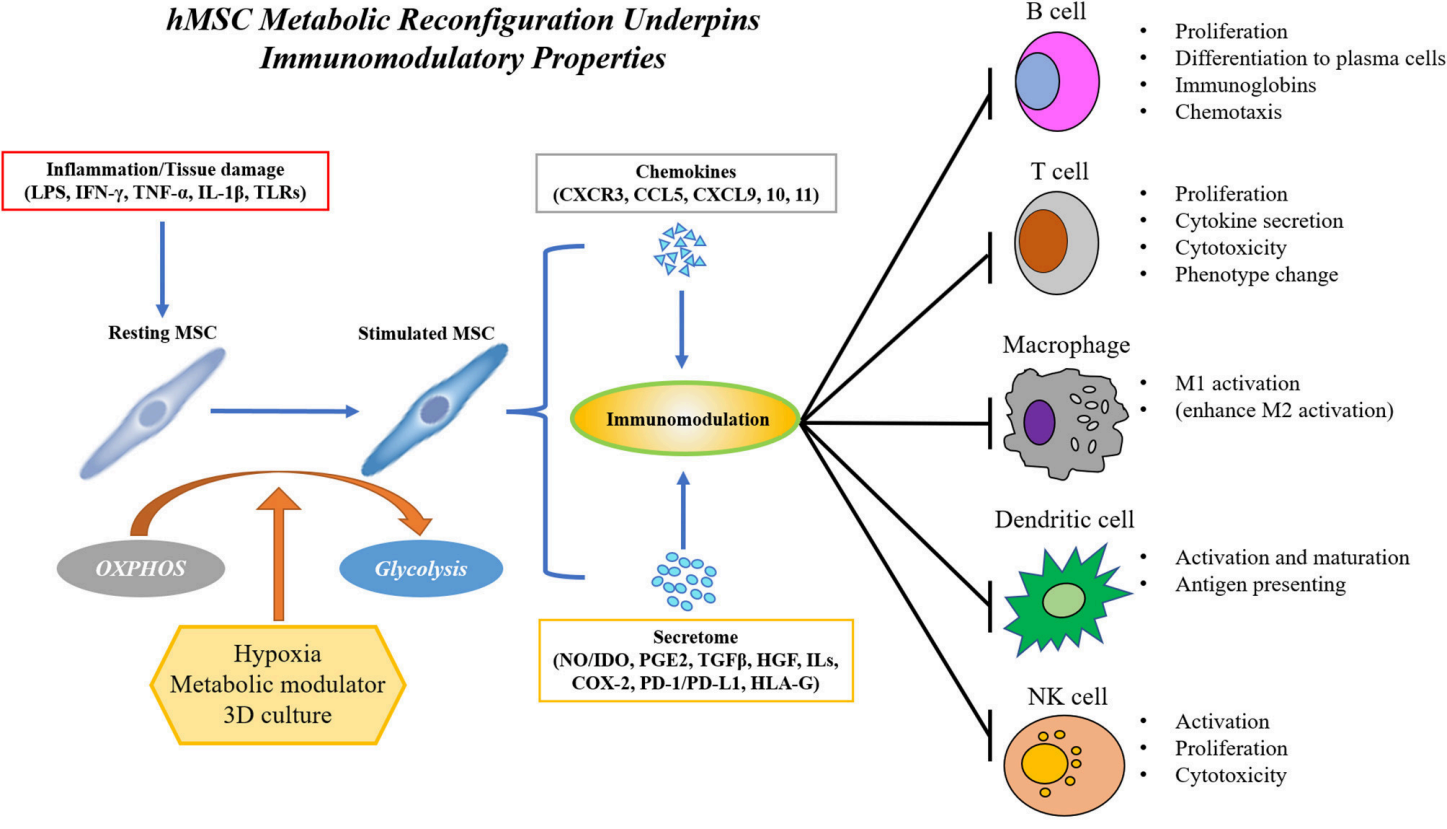
hMSC immunomodulation requires polarization by inflammatory environment and is achieved by the secretion of immunomodulatory factors such as chemokines and cytokines, extracellular vesicles and exosome, and direct cell-cell contact. hMSC's immunomodulatory property requires a metabolic reconfiguration toward aerobic glycolysis to sustain the production of secretome. hMSC's immunomodulatory capacity can be enhanced by modulation of hMSC metabolism via hypoxia, small molecule metabolic mediators, or 3D aggregation.

As mentioned earlier in this review, an attractive feature of hMSC as a cell therapy product is their immunomodulatory properties in response to environmental stimuli from surrounding tissues resulting in the secretion of beneficial cytokines and cellular components such as microRNA and extracellular vesicles; as might be expected, these properties are significantly influenced by metabolism. As shown in Figure 2, in the presence of inflammatory cytokines such as interferon-γ (IFN-γ) alone or in combination with tumor necrosis factor-α (TNF-α) or interlukin-1 (IL-1), MSCs secrete chemokines such as CXC-chemokine receptor 3 (CXCR3), CC-chemokine receptor 5 (CCR5) ligands, CXC-chemokine ligand 9 (CXCL9), CXCL10, and CXCL11 (52, 53), which attract immune cells via chemotaxis (52, 54–56). Recruited T cells are inhibited by activated hMSCs through the secretion of indoleamine 2,3-dioxygenase (IDO), a catabolic enzyme that regulates tryptophan metabolism (54). Besides IDO, hMSC is also a potent source of other soluble immunosuppressive factors, such as nitric oxide (NO, in rodent MSCs), prostaglandin E2 (PGE2), transforming growth factor-β1 (TGF-β1), hepatocyte growth factor (HGF), interleukins and cyclooxygenase 2 (COX-2). MSCs promote the polarization of macrophages from a pro-inflammatory M1 phenotype to an anti-inflammatory M2 phenotype and suppress IL-6 and TNF-α production in macrophages through secretion of PGE2 and IDO (57–61). Similarly, co-culture with MSCs inhibits the maturation and activation of antigen-presenting dendritic cells (DCs) and reduces B cell proliferation by increased production of IL-10, chemokine receptors, and immunoglobulins (62–68). Compared to B cells, MSCs inhibit T cell proliferation regardless of their lineage difference (naïve, CD4+ or CD8+ lineage) (20, 69). Moreover, MSCs from multiple sources exhibit similar effects on inducing apoptosis of T cells (70). IDO, HGF, TGF-β, PGE2, and PD-1/PD-L1 ligation from MSCs all contribute to the immunosuppressive effect (20, 71–75). As the hMSC secretome is central to their immunomodulatory properties, preserving the secretory properties of hMSC during long-term expansion has become an important engineering challenge in hMSC translation (4).

The profile of MSC secretome is tightly regulated by intrinsic (e.g., donor morbidity and aging) or external (e.g., culture conditions) factors through metabolic regulation. Adipose-derived stem cells showed a higher secretion of immunomodulatory cytokines, such as IL-6 and TGF compared to bone marrow (BM)-MSCs due to higher metabolic activity (76). Conversely, compared to cells obtained from lean patients, ASCs from obese patients exhibiting reduced glycolytic activity and upregulated expression of inflammatory genes and increased secretion of inflammatory cytokines such as IL-6 and IL-8 (30, 77, 78), making them less effective in suppressing lymphocyte proliferation and activating the M2 macrophage phenotype (79). The expression of inflammation-response genes has been reported to decline in hMSC from aged donors as they have altered metabolic profile, although conflicting results were also reported (80–82). Interestingly, recent clinical study has shown that hMSCs isolated from patient with atherosclerosis have impaired mitochondrial functional properties, contributing to the reduced suppression of T cell proliferation (33). To identify the specific role of metabolism in sustaining hMSC secretion of immunomodulatory cytokines, we observed a pronounced shift in hMSC energy metabolism toward glycolysis upon activation by IFN-γ treatment (20) and showed that inhibition of these metabolic changes prevented the production of the key immunosuppressive cytokines such as IDO and PGE2. We also demonstrated that mitochondrial ROS and Akt/mTOR signaling play a critical role in initiating this metabolic remodeling in response to inflammatory stimuli (20, 55, 83). It is not surprising that culture conditions favoring glycolysis also potentiate MSC immunomodulatory properties. As mentioned above, hypoxia culture upregulates MSC secretion of IDO, PGE2, PD-L1, ILs, etc., and enhances inhibition of CD4+/CD8+ T lymphocyte proliferation while increasing the regulatory T cells (T-reg) populations (84–86). In addition to the MSC secretome, direct cell-cell contact could directly interact with immune cells (87–92), but the influence of specific MSC metabolic profiles on these interactions remain to be investigated.

An important subset of the MSC secretome is extracellular vehicles (EVs) and exosomes, which are small membrane vesicles (ranging from 50 to 1,000 nm) derived from multi-vesicular bodies or from the plasma membrane and are enriched with proteins, lipids, and nucleic acids for intercellular communication including immune regulation (93, 94). MSC-derived EVs and exosomes have been tested in several disease models such as acute kidney injury, experimental autoimmune encephalomyelitis, type-1 diabetes mellitus and myocardial ischemic injury through microRNA (miRNA) regulation (95–99). Mechanistically, MSC-derived EVs and exosomes are enriched with miRNA such as miR-15a, miR-15b, miR-16 that inhibit CXC ligands and suppress chemotaxis of macrophage (95, 100). Recent studies have shown that metabolism regulates EV biogenesis and cargo composition through regulation of endosomal secretion pathways. In tumor cells, pyruvate kinase type M2 (PKM2), the rate limiting enzyme in glycolysis, acts as a protein kinase to promote exosome release via phosphorylating snaptosome-associated protein 23 (SNAP-23), which mediates the fusion of intracellular vesicles with membrane compartments (101). HIF-1α overexpression in hMSCs significantly enhanced exosome secretion and also upregulated Notch ligand Jagged-1, which induce dendritic cell maturation and regulatory T cell proliferation (102, 103). Hypoxia is known to mediate the expression of Rab22 and Rab20 and ceramide production, which are associated with EV formation and secretion (104). MSCs also manage intracellular oxidative stress by targeting depolarized mitochondria to the plasma membrane and unload partially depolarized mitochondria as EVs to enhance cell survival (105). Conversely, secreted exosomes regulate metabolism of recipient cells. For example, MSC exosome carry a cargo rich in active glycolytic enzymes and promote ischemic myocardium repair by enhancing glycolytic flux to compensate for the reduced OXPHOS in defective mitochondria (106). Future studies are needed to establish the mechanistic connection between hMSC metabolism and biogenesis and cargo composition of hMSC-derived EV.

## Targeting hMSC Metabolism to Enhance Immunomodulatory Properties in Preclinical Studies

Investigating the mechanistic connections between metabolism and immunomodulation has identified specific molecular targets that can be modulated to overcome metabolic deficiency due to donor age and morbidity, and to enhance the therapeutic potency of culture-expanded hMSCs. Table 1 summarized preclinical studies of enhanced MSC immunomodulation via metabolic regulation. As discussed above, hypoxia treatment is commonly used to enhance hMSC immunoregulatory properties by increasing hMSC anti-inflammatory properties while attenuating the secretion of pro-inflammatory cytokines, both *in vitro* and *in vivo* (84–86). In a preclinical study, hypoxic pre-treatment of hMSC enhanced the secretion of IL-10 and Fas ligand, thereby reducing recruitment of inflammatory cells, resulting in a more organized granulation tissue at the wound site in an excisional skin-healing mouse model (85). Transplantation of hMSC expanded under hypoxia in a humanized mouse graft vs. host disease (GVHD) model improved animal survival and weight loss, and reduced histopathologic injuries in GVHD target organs, presumably due to enhanced PGE-2 secretion and reduced IL-6/IL-8 secretion (110). To overcome the complexity and inconsistency associated with *in vitro* hypoxia culture, overexpression of HIF1-α and hypoxia mimetics targeting HIF pathway are being actively pursued to enhance hMSC immunomodulatory properties (114, 115). Oxidative preconditioning of MSCs by ROS leads to redox-dependent HIF-1α stabilization and reduced apoptosis in inflammatory environment (116, 117). Pretreatment with N-acetylcysteine (NAC), which stabilizes HIF-1, improved MSC anti-inflammation and cell retention in bleomycin-induced lung injury model by improving antioxidant capacity (118).

**Table 1:** Metabolic enhancement of mesenchymal stem cell-mediated immunomodulation in preclinical studies.

**MSC source**	**Pre-treatment**	**Metabolic targets**	**Functional enhancement**	***In vivo* study**	**References**
Rats adipose	Hypoxia	HIF	Enhance secretion of angiogenesis and neuroprotection cytokines	Improve functional recovery of DED rat; enhanced eNOS expression; increased expression of endothelial and smooth muscle markers;	(107)
Mice bone marrow	Tetrahydrocannabinol or with AM630	Mitochondrial respiration	Increased MSC IL-10 production; activated MSC ERK signaling pathway; enhance immunomodulation of microglia;	Reduced thermal hyperalgesia and mechanical allodynia response; reduced inflammation in chronic constriction injury model;	(108)
Human bone marrow	Hypoxia	HIF	Upregulated mRNA levels of IL-1β, IL-6, IL-8, and TGF β-1; mitogenic, chemoattractive and angiogenic paracrine effects	Enhance Balb/c nude mouse skin wounds healing process; Increased macrophage recruitment at wound site;	(109)
Human umbilical cord blood	Hypoxia and calcium ion	HIF	Reduced secretion of IL-6 and IL-8 and increased secretion of PGE2. Improve the inhibition of T cell proliferation.	Improve survival of humanized GVHD mouse model; decreased immune cell infiltration and characteristic tissue injuries	(110)
Human umbilical cords	IL-1β	Glycolysis	Upregulated COX-2, IL-6, IL-8 gene expression; Enhanced COX-2 protein expression;	Modulate the balance of macrophage polarization; reduced local inflammation and improve migration to DSS-induced murine colitis	(111)
Human bone marrow or umbilical cord blood	IFN-γ	Glycolysis and mitochondria	Increased gene expression of CXCL9, CXCL10, CCL8, and IDO. Enhanced secretion of IDO and inhibition of hPBMCs proliferation.	Reduced the symptoms of graft-versus-host disease (GVHD) in NOD-SCID mice and improve survival rate;	(112)
Human bone marrow	3D aggregation	Mitochondria	Increased gene expression of CXCR4, TSG-6, STC-1, IL-24, TRAIL, and LIF; elevated expression of TSG-6, LIF, and STC-1; Decreased macrophage activation	Decreased the protein content of the lavage fluid and neutrophil activity; reduced levels of the proinflammatory molecules in mouse model for peritonitis	(113)

Pre-activation of MSCs with immunomodulatory cytokines has been widely reported to enhance MSC immunomodulatory effect in various preclinical models. Interestingly, many such cytokines are also metabolic regulators and the extent of their effects is influenced by oxygen levels and MSC's metabolic plasticity (119, 120). Pretreatment of hMSC with IFN-γ activates hMSC's anti-inflammatory properties by enhancing the secretion of anti-inflammatory cytokines and inhibits the proliferation of NK cells and CD4+/CD8+ T cells (20, 111, 121, 122). Infusion of IFN-γ pretreated MSCs in an immunodeficient mouse model significantly reduced the symptoms of GVHD and improved survival (112). As mentioned above, IFN-γ treatment reconfigures hMSC metabolism toward a glycolytic phenotype, generating a metabolic profile that enhances cell survival and sustains the secretion of immunomodulatory factors (20). Fan et al. reported that MSCs preconditioned by IL-1β exposure significantly attenuated the development of dextran sulfate sodium (DSS)-induced colitis in mice by enhancing MSC migration to the inflammatory site via upregulating CXCR4 expression (111). The IL-1 cytokine family members are important regulators of metabolism and upregulate glycolysis in various cell types (119, 123). TGF- β, a pleiotropic cytokine involved in immune regulation, is also a potent regulator of hMSC immunomodulatory properties and can display either anti-inflammatory or proinflammatory effects depending on the cell niche. TGF-β activates MSC to promote immune response and altered hASC secretory profile (124–126). Interestingly, TGF-β signaling in tumor growth is compartment-specific and induces a “Warburg-like” metabolism in cancer-associated fibroblasts that fuels tumor growth; a similar metabolic shift toward glycolysis is also observed in human chondrocytes and plays important role in maintaining cartilage homeostasis (127, 128). The effects of TGF-β on hMSC metabolic properties during immune activation remains to be elucidated.

As mentioned above, 3D aggregation reconfigures hMSC metabolic state toward glycolysis and this approach has emerged as a novel engineering strategy to potentiate MSC secretory and immunomodulatory functions. Bartosh et al. reported *in vivo* aggregation of MSCs into 3D spheroid enhanced macrophage activation in mice with regulated expression of inflammation-modulating factors TSG-6, STC-1, and COX-2 (129). Zimmerman et al. demonstrated the enhanced suppression of T cells by MSC spheroids together with IFN-γ via the secretion of IDO from 3D MSC aggregates (130). Increased secretion of PGE2 and COX2 from 3D MSC aggregates converted LPS-activated macrophages into M2 phenotype with reduced production of TNF-α, IL-6, IL-23, and CXCL2 (113, 131). Intraluminal injection of MSC aggregates in mice with DSS-induced colitis reduced local inflammatory cytokines including TNF-α, IFN-γ, IL-6, and the system inflammation marker serum amyloid A (132), whereas the local expression of PGE-2 and COX-2 in mice distal colons were increased, resulting in less body weight loss and lower disease activity score (132). Inhibition of this metabolic reconfiguration in hMSC 3D aggregates reduces secretion of IDO, PGE2, COX2, IL-6, TGF-β and other anti-inflammatory cytokines (113, 129, 131, 133). These studies demonstrate the potential of 3D aggregation to potentiate hMSC immunomodulatory properties.

## Conclusions

This review shows that energy metabolism has emerged as a central hub connecting hMSC sourcing and biomanufacturing practices to core signaling pathways that regulate hMSC phenotypic properties and clinical outcome. Modulation of hMSC metabolism by specific engineering practices or metabolic modulators is an effective strategy for enhancing hMSC functional properties and improving therapeutic potency. hMSC metabolic characteristics or fitness can also be used as defining criteria to determine cellular quality in hMSC sourcing and large-scale manufacturing. As summarized in Box 1, many open questions remain in the implementation of metabolic strategies to enhance hMSC therapeutic potency in large scale manufacturing. Future studies that elucidate the signaling roles of other intermediate metabolites are needed to identify novel targets to improve hMSC clinical outcomes.

Box 1 **Current knowledge and future directions of metabolic perturbation in hMSC biomanufacturing and immunotherapy.**

**Energy metabolism in hMSC biomanufacturing and immunotherapy**

Current knowledge

- hMSC's are metabolically “plastic” and reconfigure metabolism to match divergent demands of cellular events;
- hMSC's metabolic “fitness” or the ability to adapt metabolically influences their functional properties;
- Donor morbidity and *in vitro* bioprocessing such as extended expansion and cryopreservation reduce hMSC metabolic fitness by preconditioning is an effective strategy to enhance hMSC therapeutic potency.

Open question and future direction

- What is the mechanistic link between hMSC metabolic profile and therapeutic outcome in a given disease?
- Can hMSC metabolism profile be standardized as a potency indicator as part of Critical Quality Attributes (CQA)?
- How the media composition and culture supplement such as human platelet lysate and lipid contents used in hMSC expansion influence metabolic fitness and functional properties?
- How to maintain a desired metabolic profile during large-scale expansion, harvesting, and cryopreservation (distribution and administration) in hMSC biomanufacturing?
- How does a given metabolic profile influence specific aspect of hMSC functional properties such as angiogenesis, or immunomodulation?

## Author Contributions

XY and TM organized the structure, collected references, and wrote the manuscript. TL organized and revised the manuscript. All authors contributed to manuscript revision, read, and approved the submitted version.

### Conflict of Interest Statement

The authors declare that the research was conducted in the absence of any commercial or financial relationships that could be construed as a potential conflict of interest.
